# First nationwide point-prevalence survey on healthcare-associated infections and antibiotic use in long-term care facilities, Switzerland, September 2024

**DOI:** 10.2807/1560-7917.ES.2025.30.38.2500221

**Published:** 2025-09-25

**Authors:** Domenica Flury, Emmanouil Glampedakis, Nando Bloch, Celine Gardiol, Fabian Grässli, Simone Kessler, Jacqueline Kuhn, Tanja Kurdovsky, Stefan P Kuster, Vanja Piezzi, Matthias Schlegel, Simone Toppino, Philipp Kohler

**Affiliations:** 1 Division of Infectious Diseases, Infection Prevention and Travel Medicine, Health Ostschweiz, Cantonal Hospital of St. Gallen, University teaching and research hospital, St. Gallen, Switzerland; 2 Cantonal Infection Prevention and Control Unit, Cantonal Doctor Office, Public Health Department of Canton Vaud, Lausanne, Switzerland; 3 Federal Office of Public Health, Bern, Switzerland; 4 Epidemiology, Biostatistics and Prevention Institute, University of Zurich, Zurich, Switzerland

**Keywords:** Long-term care, Switzerland, Point-prevalence survey, Healthcare-associated infection, Antibiotic use

## Abstract

**INTRODUCTION:**

Data for healthcare-associated infections (HAI) and antibiotic use in long-term care facilities (LTCF) in Switzerland are lacking but are necessary to take actions.

**AIM:**

We aimed to estimate HAI prevalence and antibiotic use and to record existing structure and process indicators in the area of infection prevention/antibiotic use in Swiss LTCF.

**METHODS:**

We invited all Swiss LTCF for this PPS in September 2024 using the adapted Healthcare-Associated Infections in European Long-Term Care Facilities (HALT)-4 protocol. The proportion of residents with HAI and systemic antibiotic treatment was calculated for a representative sample, stratified by language region and size. We assessed resident-level and institutional risk factors for HAI in all participating institutions, using random-effects logistic regression.

**RESULTS:**

We included 94 LTCF (7,244 residents), whereof 49 LTCFs (3,375 residents) belonged to the representative sample. Median age of residents in the representative sample was 87 years (range: 36–107) and 2,334 (69.2%) were female. Prevalence of HAI was 2.2% (95% confidence interval (CI): 1.7–2.7); 2.7% (95% CI: 2.2–3.3) were receiving antibiotic treatment, with highest use in LTCF in French-speaking cantons (5.9%; 95% CI: 4.2–7.5). Urinary tract (46%) and respiratory infections (20%) were most common, aminopenicillins (26%) and nitrofurantoin (19%) the most commonly used antimicrobials. The strongest independent risk factor for HAI was presence of urinary catheters (adjusted odds ratio = 2.65; 95% CI: 1.71–4.11).

**DISCUSSION:**

Prevalence of HAI and antibiotic use in Swiss LTCFs were comparable to the European average from 2023/24. There are regional differences in antibiotic consumption. Urinary catheterisation, potentially modifiable, was the most important risk factor for HAI.

**Key public health message**

**What did you want to address in this study and why?**

Before this study, representative data on the prevalence of healthcare associated infections (HAI) and antibiotic use in Swiss long-term care facilities (LTCF) were not available. We wanted to close this knowledge gap, identify risk factors for HAI, and lay the foundation for future activities in this neglected patient setting.

**What have we learnt from this study?**

This study was done in 7,244 residents of 94 Swiss LTCFs. Similar to European data from 2023 and 2024, about one in 45 residents (2.2%) had a healthcare-associated infection. Urinary tract infections were most common, and presence of a urinary catheter was the single most important factor associated with HAI. Antibiotics were given to almost one in 40 (2.7%) residents, with antibiotic use significantly higher in the French-speaking regions.

**What are the implications of your findings for public health?**

Outside of the respiratory season, measures to reduce the burden of HAI in Swiss LTCF should primarily aim to reduce urinary tract infections. Our data also suggest that antibiotic stewardship activities will potentially have the highest impact if implemented in LTCFs in French-speaking regions.

## Introduction

Healthcare-associated infections (HAI) are among the major complications of modern medicine and represent a considerable burden in terms of both morbidity and mortality [1,2]. In a prospective cohort study performed in long-term care facilities (LTCFs) in nine European countries, 57% of residents experienced at least one HAI over the course of a year, with 4.3% of HAI leading to hospitalisation and 4.1% of HAI leading to death [3]. Already in 2009, the European Centre for Disease Prevention and Control (ECDC) launched the programme Healthcare-Associated Infections in European Long-Term Care Facilities (HALT) with the aim of developing a standard methodology to assess the HAI prevalence and the status of infection prevention and control (IPC) activities in European LTCFs. Furthermore, the HALT programme addresses antibiotic use, which is among the main drivers of antimicrobial resistance [4]. Using the HALT-4 protocol, the ECDC performed a point-prevalence survey (PPS) in countries of the European Union (EU) and the European Economic Area (EEA) in 2023 and 24, which showed an HAI prevalence of 3.1% (infections associated with the respective institution) [5]. Prevalence varied substantially between countries, ranging from 0.9% in Germany to 6.0% in Portugal. The most commonly encountered HAIs were urinary tract infections (UTI), respiratory tract infections, and skin infections. Moreover, 4.1% of LTCF residents were receiving antibiotics at the time of the PPS [5].

Surveillance of HAI and antibiotic use is essential when determining the burden of disease and informing healthcare authorities regarding potential IPC or antimicrobial stewardship interventions. In Switzerland, not a member state of the EU/EEA, no national PPS in LTCF had been conducted so far. Previous data from a convenience sample of LTCFs from the eastern and western part of the country suggest that HAI prevalence and antibiotic use might be lower than the European average [6]. Furthermore, studies on risk factors for HAI in LTCF residents are, in contrast to acute care patients, scarce. In particular the impact of institutional factors, such as the proportion of single rooms or the nurse-to-resident ratio, remains largely unknown.

Considering these knowledge gaps and with the aim of creating a data basis for the development of national minimum requirements in the area of IPC, we performed a national PPS on HAI and antibiotic use in residents of Swiss LTCF. This is in line with the national strategy for the prevention of nosocomial infections in acute and long-term-care facilities. In addition, we aimed to detect institutional and resident-level characteristics associated with HAI in order to identify novel and potentially modifiable risk factors for infection.

## Methods

### Setting, study design and long-term care facility recruitment

In Switzerland (population size 9 million in June 2024), there are ca 1,500 LTCFs, almost half of them (47%) privately owned, caring for 155,000 people annually. The mean age at admission is 82 years, with slight differences between the 26 administrative subdivisions (i.e. cantons) [7]. This PPS was performed in September 2024. We adopted two approaches for LTCF recruitment. Sample A (representative sample): in analogy to the HALT-4 PPS [8], we formed a sample representing LTCFs in Switzerland by randomly selecting 122 institutions across the country from the list of the Swiss Federal Office of Statistics (as in January 2024) [9]. Random selection was performed in such a way that institutions from different language regions were represented proportionally to the population size of each region; similarly, the number of beds of the selected institutions were representative of the overall bed size distribution of Swiss LTCFs. This number was based on a target sample size of 3,480 residents (calculated to achieve 1% precision in the estimation of national HAI prevalence as described in Suetens et al. [10]), as well as a mean number of 58 participants per institution and an expected response rate of 50%. In January 2024, emails were sent to these institutions describing the aim and content of the study along with the call for study participation. Non-responding institutions were reminded once by email and once by phone call. A financial incentive of 10 Swiss Francs (ca EUR 10) per included resident was offered to institutions of the representative sample. Because of a lower-than-expected response rate, an additional 15 randomly chosen institutions were contacted in a second recruitment phase in April 2024.

Apart from this random sample, sample B (interested sample) consisted of all other LTCF from the list of the Swiss Federal Office of Statistics. Therefore, we asked cantonal authorities and national LTCF umbrella organisations to disseminate the invitation for study participation within their networks in March 2024. No financial reimbursement was offered to this group of institutions. All participating institutions gave their written informed consent; the local ethics committee approved the study.

### Study procedures

Between July and August 2024, online educational sessions in the three national languages were held to instruct LTCF representatives on how to correctly fill in the questionnaires of the adapted ECDC HALT-4 protocol. A detailed manual was produced in three national languages and a section of frequently asked questions was continuously updated on the study homepage.

To avoid the viral respiratory season, as suggested by HALT, we performed data collection between 9 and 27 September 2024. Institutions were allocated to a particular study week; most institutions collected the data within 1–2 days. We included all residents present in the LTCF at 8:00 on the day of the PPS. The LTCF residents were required to be full-time residents, i.e. living 24 h a day in the institution. Residents who denied participation or who were temporarily absent were excluded. Data were collected from chart reviews and then entered directly into a REDCap database either by the institutional representatives or by a member of the study team (after the institution had provided the pseudonomised data). To ensure data quality, all residents with suspected HAI were discussed with a dedicated medical person from the study team or cantonal physician.

### Definitions and protocol

We used the questionnaires of the HALT-4 protocol, which consists of an institutional questionnaire (asking about structural characteristics such as size or number of healthcare personnel, but also about IPC activities, guidelines and indicators such as vaccination rate or use of hand hygiene alcohol) and a resident-level questionnaire (asking about health characteristics and care dependency, about HAI and antibiotic use). We made the following adaptions to the ECDC questionnaire: for the institutional questionnaire, we created a new variable calculating the sum of available IPC activities and guidelines for every institution. Also, we calculated the proportion of auxiliary nurses as percentage of all nursing full-time equivalents (FTE) per institution. For the resident-level questionnaire, we added a question on use of proton pump inhibitors (PPI), which has been previously shown to be associated with HAI and antibiotic resistance [11,12]. Different from the HALT-4 protocol, risk factors (wounds, dementia, catheters, urinary incontinence, etc.) were collected on a resident level for all eligible residents and not only for those with HAI. The content of the institutional and the resident-level questionnaires are appended in Supplementary Tables S1 and S2. We used the ECDC definitions for HAI, as documented in the HALT-4 protocol [8]. An HAI was defined as active when symptoms were present or when the resident was still receiving therapy for the HAI on the day of the PPS.

### Statistical analysis

To better estimate potential selection bias, we compared institutional characteristics of LTCF from the random sample who agreed against those who declined to participate in this study. For participating institutions, we summarised the answers given in the institutional questionnaire. For categorical variables, we report numbers and percentages, whereas for continuous variables, we calculated the median and the range or interquartile range (IQR) . Owing to cultural and structural differences in the management of LTCFs, results were stratified by language region, i.e. French-speaking (cantons of Fribourg, Genève, Jura, Neuchâtel, Valais and Vaud), Italian- speaking (Ticino) and German-speaking (all other cantons) regions.

We used descriptive statistics to compare the institutional and resident-level characteristics of the representative sample (see above) against the full sample of participating LTCFs. The proportion of residents with HAI and with antibiotic use was calculated for the representative and for the full sample; 95% confidence intervals (CI) around the estimates were calculated using the normal approximation method. Results were again stratified by language region.

For the risk factor analysis, we used the full sample and logistic regression to model the association of each variable with risk of HAI. Odds ratios (OR) and 95% CI are reported. Factors demonstrating statistical significance (i.e. p value < 0.05) in univariable analysis were included in a multivariable model to calculate adjusted odds ratios (aOR) and 95% CI. Because convergence issues occurred when institutions were included in the models as random effects, we primarily present results from models without random effects. After centering and rescaling some of the numeric variables, we also fitted mixed-effects (random intercepts) models accounting for institutional clustering, both for univariable and multivariable analysis, which are presented as sensitivity analyses. We used statistical software R, version 4.4.2 (Vienna: R Foundation for Statistical Computing), for all analyses. Statistical significance was considered for p values < 0.05. The lme4 package, version 1.1–35.5, was used for mixed-effects models [13].

## Results

### Institutional characteristics

Of the 141 randomly selected and invited institutions, 49 (35%) agreed to participate, constituting the representative sample. Characteristics of participating and non-participating institutions are appended in Supplementary Table S3; participating institutions were larger, had higher dependency on care among residents, and institutions from the German-speaking regions were underrepresented. In addition to the 49 institutions from the random sample, 45 LTCFs volunteered to participate, resulting in a full sample of 94 LTCFs. 

**Figure 1:**
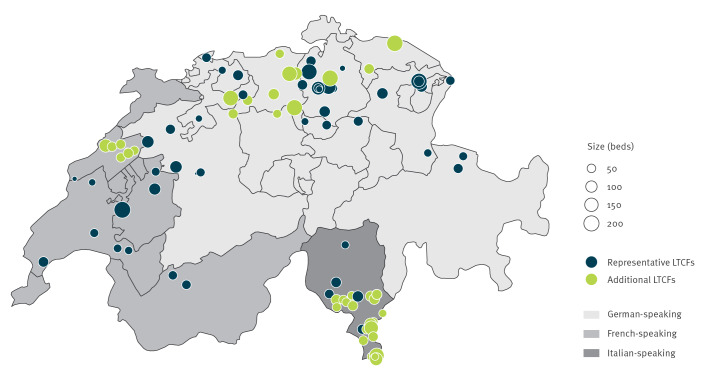
Participating long-term care facilities in Switzerland, Switzerland, 2024 (n = 94)

Of the representative sample, 31 were located in the German-, six in the Italian- and 12 in the French-speaking region; median bed size was 63 (IQR: 44–84) (Figure 1). The majority (29/49) of institutions identified themselves as general nursing homes, whereas nine of 49 identified as residential home (i.e. residents with only minimal assistance in activities of daily living). The number of registered nurses per 100 beds was 23 (interquartile range (IQR): 19–29); the median FTE of IPC professionals per 100 beds for institutions with an IPC professional was 13% (IQR: 9–40). Residents received medical care from their general practitioner (21/49 of institutions), from a facility-employed physician (10/49) or a combination of both (18/49). A median of 19% (IQR: 10–28) of HCW were vaccinated against seasonal influenza in 2023/24. 

LTCF: long-term care facility.

Institutional characteristics were similar between the representative and the full sample, except that in the full sample, more LTCF from the Italian-speaking region were included (Table 1). Comparing institutional characteristics between language regions showed that in the French-speaking region, more institutions were served by a facility-employed physician, the number of qualified nurses per 100 beds was lower, whereas the number of auxiliary nurses and the number of IPC nurses per 100 beds was higher than in the German- or Italian speaking regions. Self-reported influenza vaccination rates of both residents and HCW tended to be higher in the French- or Italian speaking region. The detailed results can be accessed in Supplementary Table S4.

**Table 1:** Institutional characteristics of the representative and the full sample of long-term care facilities, Switzerland, 2024 (n = 94)

Baseline characteristics	Representative sample n = 49		Full sample n = 94		p value
	n^a^	%^a^	n^a^	%^a^	
Language region					
French-speaking	12	24.5	18	19.2	< 0.05
Italian-speaking	6	12.2	33	35.1	
German-speaking	31	63.3	43	45.7	
Type of facility					
Residential home	9	18.4	26	27.7	0.47
General nursing home	29	59.2	50	53.2	
Mixed/other	11	22.4	18	19.2	
Facility indicators					
Nursing FTE/100 beds, median (IQR)	23 (19–29)		23 (18–30)		0.25
Auxiliary nurse FTE/100 beds, median (IQR)	27 (23–37)		31 (23–44)		0.21
Auxiliary nurses, median % (IQR)^b^	58 (49–62)		58 (49–64)		0.56
Number of beds, median (IQR)	63 (44–84)		69 (53–96)		0.23
Single rooms, median % (IQR)	91 (75–100)		90 (75–100)		0.28
Physician in charge					
Personal family physician alone	21	42.9	42	44.7	0.80
Employed by the facility alone	10	20.4	15	16.0	
Both	18	36.7	37	39.3	
Vaccinations					
Influenza^c^ vaccination residents, median % (IQR)	70 (50–80)		70 (55–85)		0.37
Influenza^c^ vaccination HCW, median % (IQR)	19 (10–28)		17 (10–28)		0.82
SARS-CoV-2^d^ vaccination residents, median % (IQR)	85 (75–90)		82.5 (70–90)		0.71
SARS-CoV-2^d^ vaccination HCW, median % (IQR)	70 (20–81)		70 (17–89)		0.72
IPC structures and parameters					
IPC-trained HCW in facility	28	57.1	65	69.1	0.21
IPC staff FTE/100 beds, median % (IQR)	13 (9–40)		13 (8–36)		0.76
Alcoholic hand rub use, median litres/100 beds (IQR)^e^	313 (186–427)		297 (183–468)		0.79
Surveillance of HAI in place	7	14.3	16	17.0	0.86
IPC committee in place	14	28.6	37	39.4	0.27
IPC activities					
Median number of all activities (IQR)	8 (7–9)		8 (7–10)		0.46
IPC teaching for nurses and paramedics	37	75.5	65	69.1	0.55
IPC teaching for physicians	4	8.2	13	13.8	0.47
Development of nursing standards	46	93.9	86	91.5	0.86
Surveillance of residents with multiresistant pathogens	32	65.3	68	72.3	0.50
Dedicated person for outbreak reporting/management	41	83.7	84	89.4	0.48
Reporting of surveillance results to nurses/physicians	20	40.8	50	53.2	0.22
Control on reprocessing of medical devices/equipment	26	53.1	56	59.6	0.57
Decision on isolation measures for residents with MDRO	43	87.8	86	91.5	0.68
Organisation/control/reporting of audits on IPC measures	45	91.8	80	85.1	0.72
Organisation/control/reporting of hand hygiene measures	45	91.8	80	85.1	0.38
Possibility for residents to get the influenza vaccine	49	100	94	100	1
Possibility for residents to get the SARS-CoV-2 vaccine	42	85.7	86	91.5	0.43
IPC guidelines					
Median number of all guidelines (IQR)	6 (5–7)		6 (5–8)		0.44
Management of multiresistant pathogens	41	83.7	84	89.4	0.48
Hand hygiene	49	100	94	100	1
Management of urinary catheters	40	81.6	75	79.8	0.97
Management of vascular catheters	19	38.8	49	52.1	0.18
Management of feeding tubes	25	51.0	53	56.4	0.66
Management of respiratory virus outbreaks	46	93.9	90	95.7	0.93
Management of gastrointestinal outbreaks	43	87.8	81	86.2	1.0

FTE: full-time equivalent; HAI: healthcare-associated infection; HCW: healthcare worker; HH: hand hygiene; IPC: infection prevention and control; IQR: interquartile range; MDRO: multidrug-resistant organism; SARS-CoV-2: severe acute respiratory syndrome coronavirus 2.

^a^ If not stated otherwise.

^b^ Percentage auxiliary nurse FTE of total nursing FTE.

^c^ Season 2023/24.

^d^ Any SARS-CoV-2 vaccination.

^e^ For the year 2023; missing data for 31 (representative sample) and 49 (full sample) institutions.

### Characteristics of residents

We included 7,244 residents, thereof 3,375 (47%) in the representative sample. In the representative sample, median age was 87 years (IQR: 81–91) and 2,334 (69%) were female. A urinary catheter was present in 6% of residents, 65% had incontinence and 57% were disoriented, 32% were non-mobile. Of note, 37% of residents were under PPI treatment at time of the PPS, ranging from 0 to 71% across institutions. Resident characteristics, except language region distribution, were again similar between those in the representative and those in the full sample (Table 2).

**Table 2:** Resident characteristics in the representative and the full sample of long-term care facilities, Switzerland, 2024 (n = 7,244)

	Representative sample n = 3,375		Full sample n = 7,244		p value
	n^a^	%	n^a^	%	
Median age in years (range)	87 (36–107)		87 (32–107)		0.11
Median number of years in institution (range)	2 (0–31)		2 (0–57)		< 0.01
Median care dependency score^b^ (IQR)	7 (5–9)		7 (5–9)		< 0.01
Sex					
Female	2,334	69.2	5,056	69.8	0.52
Male	1,041	30.8	2,188	30.2	
Language region					
German	2,250	66.7	3,660	50.5	< 0.001
French	767	22.7	1,232	17.0	
Italian	358	10.6	2,352	32.5	
Medical history					
Hospital stay in the last 3 months	379	11.2	730	10.1	0.08
Surgery in the last 30 days	64	1.9	142	2.0	0.88
Mobility					
Ambulant	2,296	68.0	4,583	63.3	< 0.001
Wheelchair	997	29.6	2,485	34.3	
Bedridden	82	2.4	176	2.4	
Risk factors					
Urinary catheter	215	6.4	473	6.5	0.79
Vascular catheter	16	0.5	48	0.7	0.30
Temporal and/or spatial disorientation	1,927	57.1	4,262	58.8	0.09
Incontinence (urinary and/or faecal)	2,200	65.2	4,929	68.0	< 0.01
Proton pump inhibitor	1,233	36.5	2,856	39.4	< 0.01
Decubital ulcer	147	4.4	322	4.4	0.87
Other chronic wounds	422	12.5	876	12.1	0.57

IQR: interquartile range.

^a^ If not stated otherwise.

^b^ Full score ranging from 0 (< 20 min of care per day) to 12 (> 220 min of care per day).

### Prevalence of healthcare-associated infections and antibiotic use

**Figure 2:**
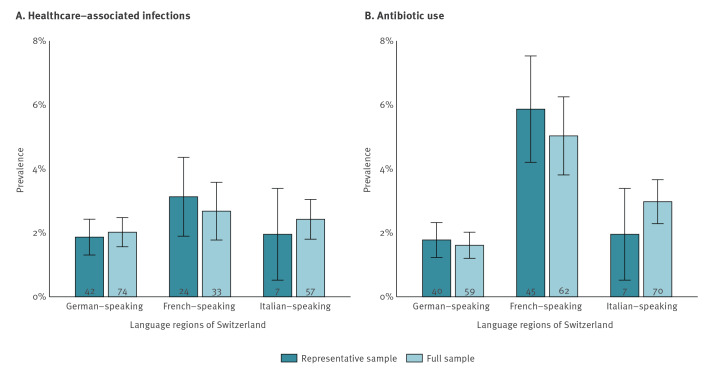
Prevalence of healthcare-associated infections and of antibiotic use in long-term care facilities, representative and the full sample, by language region, Switzerland, 2024 (n = 72,44)

**Figure 3:**
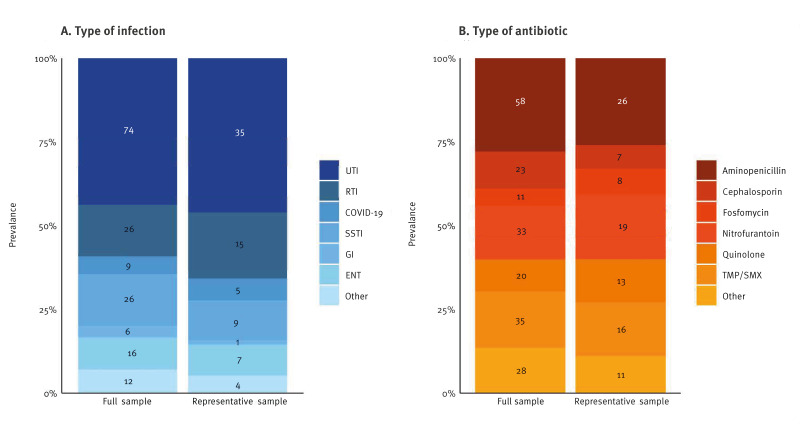
Frequencies of healthcare-associated infections and of antibiotic substances in residents of Swiss long-term care facilities in the representative and the full sample, Switzerland, 2024 (n = 7,244)

GI: gastrointestinal infection; ENT: ear-nose-throat infection; RTI: respiratory tract infection; SSTI: skin and soft-tissue infection; UTI: urinary tract infection; TMP/SMX: trimethoprim-sulfamethoxazole.

On the day of the PPS, 75 HAIs were found in 73 of 3,375 residents of the representative sample, corresponding to a prevalence of 2.2% (95% CI: 1.7–2.7); the respective number was similar in the full sample, with a HAI prevalence of 2.3% (95% CI: 1.9–2.6). The HAI prevalence was similar between the different language regions (Figure 2), ranging between 0% and 11% across institutions, with the detailed numbers accessible in Supplementary Figure S1. The most common were UTI (46%), followed by respiratory infections (20%) and skin/soft tissue infections (12%) (Figure 3). At least one pathogen was reported in 35 of 75 HAI (47%, total of 43 pathogens), with *Escherichia coli* (9/43) and severe acute respiratory syndrome coronavirus 2 (SARS-CoV-2; 5/43) being the most common. For the remainder of the HAIs, pathogen was not reported, probably because of missing culture or swab. Details on HAI types and identified pathogens are appended in Supplementary Table S5.

Numbers at the bottom of the bars represent the number of residents with at least one healthcare-associated infection (A) or receiving at least one systemic antibiotic (B). Whiskers represent 95% confidence intervals around the point estimates.

In the representative sample, 92 residents received antibiotic treatment, corresponding to a prevalence of 2.7% (95% CI: 2.2–3.3); in the full sample, the respective prevalence was 2.6% (95% CI: 2.3–3.0). Antibiotic use was more common in the French (5.9%; 95% CI: 4.2–7.5) compared with the Italian (2.0%; 95% CI: 0.5–3.4) or German-speaking regions (1.8%; 95% CI: 1.2–2.3) (Figure 2). Antibiotic use ranged between 0% and 27% across institutions; the detailed numbers are available in Supplementary Figure S1. Overall, 66% of antibiotic courses were given as therapy and 34% for prophylactic indication. Aminopenicillins (26%), trimethoprim/sulfamethoxazole (16%) and nitrofurantoin (19%) were the most commonly used antibiotic substances (Figure 3). Among the 49 residents not matching any HAI criteria, 28 received substances, either as prophylaxis or therapy, which are usually given for UTIs, such as trimethoprim/sulfamethoxazole (n = 11), nitrofurantoin (n = 10) or fosfomycin (n = 7).

### Factors associated with healthcare-associated infections

Institutional and resident-level factors shown in Table 1 were tested in univariable analysis; these results are available in Supplementary Table S6. In multivariable analysis, presence of urinary catheters (aOR = 2.65: 95% CI: 1.71–4.11), incontinence (aOR = 1.75; 95% CI: 1.12–2.74), chronic wounds (aOR = 1.68; 95% CI: 1.16–2.44) and recent hospitalisation/surgery (aOR = 1.64; 95% CI: 1.08–2.49) were independently associated with HAI (Table 3). Results of the sensitivity analysis accounting for institutional clustering are appended in Supplementary Table S8 and yielded similar results. The only institutional characteristic associated with HAI both in the fixed and the mixed-effects model was FTE of auxiliary nurses per 100 beds (aOR per % increase = 1.02; 95% CI: 1.01–1.04). However, the proportion of auxiliary nurses among all nursing staff was not associated with the outcome (only tested in univariable analysis).

**Table 3:** Multivariable logistic regression regarding presence of healthcare-associated infections in long-term care residents (full sample), Switzerland, 2024 (n = 164)

	aOR	95% CI	p value
Resident-related factors			
Male sex	1.22	0.87–1.70	0.25
Care dependency^a^, median (IQR)	1.00	0.93–1.07	0.89
Disorientation	1.11	0.77–1.59	0.58
Wheelchair/bedridden	1.36	0.94–1.95	0.10
Urinary catheter	2.65	1.71–4.11	< 0.001
Incontinence	1.75	1.12–2.74	0.01
Chronic wound/decubital ulcer	1.68	1.16–2.44	0.006
Hospitalisation/surgery last 3 months/30 days	1.64	1.08–2.49	0.02
Institutional factors			
Nursing FTE/100 beds, median (IQR)	1.01	0.99–1.02	0.39
Auxiliary nurse FTE/100 beds, median (IQR)^b^	1.02	1.01–1.04	0.002
Physician in charge			
Personal family physician alone	Reference		
Employed by the facility alone	1.64	1.03–2.62	0.04
Both	1.40	0.99–2.00	0.06
IPC structures and parameters			
IPC committee in place	1.38	0.98–1.93	0.06

aOR; adjusted odds ratio; CI: confidence interval; FTE: full-time equivalent; IPC: infection prevention and control; IQR: interquartile range.

^a^ Full score ranging from 0 (< 20 min of care per day) to 12 (> 220 min of care per day).

^b^ Not included in the multivariable model because of high number of missing data (n = 3,049).

Raw numbers are available in Supplementary Table S7.

## Discussion

In this first nationwide and representative PPS in Swiss LTCF, the prevalence of HAI was 2.2% and antibiotic treatment was given to 2.7% of residents. Urinary tract infections were most common, and presence of a urinary catheter was the most important and potentially modifiable risk factor for HAI. These data may inform healthcare providers and public health authorities regarding future IPC and antibiotic stewardship interventions in this particular setting.

The HAI prevalence of 2.2% is comparable to the European average. In the last HALT-4 PPS performed in 2023 and 2024, overall HAI prevalence in more than 66,000 residents across LTCFs in Europe was 3.1% with substantial geographical variation [10]. Older data (collected before 2007) from the United States (US) showed a considerably higher prevalence of 12%, although the methodology used was different from the European studies [14]. The distribution of the most common infections in our study was similar to the European and the US data, with UTI, respiratory and skin infections the most common [10,14]. Of note, 7% of our HAI were due to COVID-19, whereas in the European study, the corresponding figure was 2.7%; the US study was performed before the COVID-19 pandemic.

Presence of a urinary catheter was the most important factor associated with HAI in our study. Several other studies identified urinary catheters as important risk factors for HAI in the LTCF population [14-18]. Although causality cannot be inferred from our data, these findings clearly point to the area with the greatest potential for improvement in the long-term care setting, the prevention of UTI. This has been nicely shown in a large-scale nationwide project in the US, which demonstrated an almost 50% reduction of the incidence of catheter-associated UTIs in nursing homes after implementation of a technical and a socio-adaptive bundle of measures aiming to improve management of urinary catheters [19]. Interestingly, most institutional factors in our analysis were not associated with HAI, which is in line with a multi-level modelling study based on the ECDC HALT-3 data where the only institutional factor associated with decreased HAI was giving feedback on surveillance results to LTCF staff, but not the percentage of single rooms or the presence of an IPC committee in the institution [17]. In contrast, studies focusing on viral respiratory infections have often identified LTCF-level factors, such as infection prevention measures [20,21], ward size and type of air circulation [22], or duration of dedicated resident care [23], to be associated with infection. The only institutional variable associated with the outcome in both the fixed and the mixed effects model was the number of FTE of auxiliary nurses. We think that this is rather a reflection of the intensity of care and not of the quality of care, as the ratio of auxiliary nurses among all nursing staff was not associated with HAI. We would like to emphasise that socioeconomic factors such as index of deprivation in a certain geographic area have previously been linked to the occurrence of HAI [24]. We did, however, not study these factors.

Antibiotic use was 2.7% in our population, which is lower than the 4.1% reported in the European data, but comparable to other central European countries (Germany 1.2%, Italy 3.2%, France 2.6%) [5]. Aminopenicillins were the most frequently used antibiotic class. In line with UTI being the most common infection, trimethoprim/sulfamethoxazole and nitrofurantoin were also commonly administered. Quinolones were less commonly prescribed than in previous studies [6], potentially reflecting changes in national guidelines on UTI treatment. We observed substantial differences in antibiotic use between language regions, the French-speaking region showing the highest antibiotic consumption. Given the similar HAI rates between regions, these results suggest that the threshold to prescribe antibiotics might be lower in French-speaking cantons, a hypothesis which has been raised previously, not only for the LTCF setting [6] but also for the outpatient setting [25]. Besides cultural differences between regions, which were not captured with this survey, we identified certain structural differences between language regions, such as higher percentage of facility-employed physicians and lower number of qualified nurses per 100 beds in French-speaking regions. These factors might also have contributed to the different prescribing patterns between regions.

A third (33%) of antibiotic treatments were prescribed for prophylaxis, which is in line with the European data from 2023 and 24 with 29% prophylaxis [26]. This high number potentially represents a topic for future antibiotic stewardship interventions. Of note, 57% of residents receiving antibiotics without documented HAI were given trimethoprim/sulfamethoxazole, nitrofurantoin or fosfomycin as prophylaxis or therapy. Because these substances are almost exclusively used against UTI, these data suggest overdiagnosis and overtreatment for this particular indication. Indeed, asymptomatic bacteriuria is a common cause of antibiotic overtreatment in this population [27]. For instance, in a survey among US nursing home personnel, only 28% knew that cloudy or smelly urine should not be routinely cultured [28]. Strikingly, antibiotic use was very skewed in our study, as few institutions were responsible for the large part of antibiotic treatments. Accordingly, antibiotic – and most probably also diagnostic – stewardship interventions will potentially have the highest impact if tailored to such institutions with potentially high unnecessary antibiotic prescriptions.

It has previously been shown that PPI not only increase the risk for *Clostridioides difficile* infections, but also the risk for colonisation with resistant Enterobacterales [12,29]. Our study did not primarily assess these outcomes, and PPI use was not associated with HAI in our analysis. Nevertheless, we found that 39% of all LTCF residents were treated with PPI, with considerable variation between institutions. These findings question the accuracy of PPI indications and support the recommendation that stewardship interventions should also aim to improve the use of PPI, particularly in the LTCF population [12].

The large sample size and our attempt to assess the association of both institutional and resident-level factors with HAI are the main strengths of the study. Further, the consistency of the estimates between the representative and the full sample underlines the external validity of our data. Our data are in line with the European point-prevalence data and the recently published longitudinal data for HAI in LTCF, confirming the high incidence and burden of HAI in LTCF [3,5]. Our study also has limitations. Firstly, the representativeness of our random sample can be questioned. Only 36% of invited institutions agreed to participate; furthermore, incentivising institutions with a financial compensation could have introduced further bias. However, given that the estimates between the representative and the full sample were fairly similar, we believe that our results appropriately mirror the epidemiology of HAI and antibiotic use in Swiss LTCF. If at all, our results are biased towards overestimation of HAI prevalence, as factors associated with higher HAI prevalence (high care of dependency score, institutions from French/Italian-speaking cantons) were overrepresented among participating institutions. Secondly, the study was deliberately performed outside of the respiratory season. This must be taken into account when interpreting our data. It is well known that viral respiratory infections such as influenza, COVID-19 or infections with respiratory syncytial virus is common in this population [3,21]. Of note, only an estimated 19% of HCW were vaccinated against influenza in our sample. Given the potential protective effect of HCW vaccination against influenza in long-term care residents [30], this may indicate another approach to reduce the burden of HAI in Swiss LTCFs. Thirdly, some – with regard to HAI – important baseline variables such as comorbidities or immunosuppression were not part of our resident questionnaire. However, we think that other variables such as age, level of care dependency, incontinence or recent acute care contact can serve as reasonable proxies for these variables. Fourthly, due to limited resources, no systemic validation was carried out, but focus was placed on good training and support during data collection. Finally, we do not know whether placement of urinary catheters or urinary incontinence occurred before the onset of infection. However, placement of a urinary catheter is usually not part of the recommended UTI management in this population, which is why we think that the catheter was mostly in place before the infection. Also, we do not know whether indications for urinary catheter placement were correct.

## Conclusions

We believe that our data close an important knowledge gap regarding the burden of HAI and antibiotic use in Swiss LTCF. Future interventions in this setting should ideally combine antibiotic and potentially also diagnostic stewardship elements with IPC measures with a special focus on the prevention of UTI. The large variation in antibiotic use between institutions could be leveraged to optimise the cost-effectiveness of future antibiotic stewardship interventions.

## Data Availability

The original data can be solicited from the authors upon reasonable request.

## Supplementary Data

449000 Supplement
