# Supplementary material for: First nationwide point-prevalence survey on healthcare-associated infections and antibiotic use in long-term care facilities, Switzerland, September 2024
Source: Euro Surveill. 2025 Sep 25;30(38):2500221. doi: 10.2807/1560-7917.ES.2025.30.38.2500221 (PMC12475890; doi:10.2807/1560-7917.ES.2025.30.38.2500221)

Supplementary material

This supplementary material is hosted by *Eurosurveillance* as supporting information alongside the article “First nationwide point-prevalence survey on healthcare-associated infections and antibiotic use in Swiss long-term care facilities”, on behalf of the authors, who remain responsible for the accuracy and appropriateness of the content. The same standards for ethics, copyright, attributions and permissions as for the article apply. Supplements are not edited by *Eurosurveillance* and the journal is not responsible for the maintenance of any links or email addresses provided therein

Supplementary Tables

Table S1. Institutional questionnaire (in German), adapted from the HALT-4 protocol, Switzerland, 2024.

Field Label	Choices, Calculations, OR Slider Labels
Datum Institutionsfragebogen	
Art der Einrichtung	1, Altersheim | 2, Pflegeheim | 3, Gerontopsychiatrie | 4, Gemischt | 88, Andere Einrichtung
Gemischt: Bitte spezifizieren.	
Andere Einrichtung: Bitte spezifizieren.	
Ist qualifizierte Pflege 24 von 24 Stunden in der Einrichtung verfügbar?	1, Ja | 0, Nein
Welches Instrument verwendet Ihre Institution zur Bewertung der Pflegebedürftigkeit?	1, RAI Score | 2, BESA Score | 3, Score PLAISIR
Gesamtzahl der Vollzeitäquivalente von qualifiziertem Pflegepersonal	
Gesamtzahl der Vollzeitäquivalente von Pflegehilfen	
Gesamtzahl der Bewohnendenzimmer	
Gesamtzahl der Einzelzimmer	
Gesamtzahl der Einzelzimmer mit eigenem Badezimmer (Toilette und Waschbecken und/oder Dusche)	
Betten in der Einrichtung (belegte und nicht belegte Betten)	
Belegte Betten zum aktuellen Zeitpunkt	
Die medizinische Versorgung, einschließlich der Verschreibung von antimikrobiellen Substanzen, erfolgt in der Einrichtung:	1, Ausschließlich durch persönliche Hausärzt*innen oder (hausärztliche) Gemeinschaftspraxen | 2, Ausschließlich durch in der Einrichtung angestelltes medizinisches Personal (Heimärzt*in) | 3, Kombination aus beiden Versorgungsformen
Werden die medizinischen Massnahmen in der Einrichtung durch eine(n) bestimmte(n) Ärzt*in koordiniert?	0, Nein, es gibt keine interne oder externe Koordination der medizinischen Maßnahmen. | 1, Ja, ein(e) in der Einrichtung angestellte(r) Ärzt*in (intern) koordiniert die medizinischen Maßnahmen. | 2, Ja, ein(e) nicht in der Einrichtung angestellte(r) Ärzt*in (extern, z.B. ein(e) Hausärzt*in) koordiniert die medizinischen Massnahmen. | 3, Ja, sowohl ein(e) in der Einrichtung angestellte(r) als

	auch ein(e) externe(r) Ärzt*in koordinieren die medizinischen Massnahmen.
Geschätzter Prozentsatz der gegen saisonale Influenza geimpften Bewohnenden (%)	
Geschätzter Prozentsatz der gegen saisonale Influenza geimpften Mitarbeitenden (%)	
Geschätzter Prozentsatz der gegen Sars-CoV2 geimpften Bewohnenden (%)	
Geschätzter Prozentsatz der gegen Sars-CoV2 geimpften Mitarbeitenden (%)	
Gibt es in der Einrichtung in Infektionsprävention und -kontrolle geschulte Personen, die dem Pflegepersonal unterstützend zur Verfügung stehen?	1, Ja | 0, Nein
Wenn ja, diese Person/en ist/sind:	1, Link-Nurse oder Fachexpert*in Infektionsprävention | 2, Ärzt*innen | 3, Sowohl als auch (Ärzt*in und Link-Nurse/Fachexpert*in Infektionsprävention) | 88, Andere Person
Andere Person: Bitte spezifizieren.	
Diese Person/en:	1, Arbeitet/arbeiten in der Einrichtung (intern) | 2, Ist/sind nicht in der Einrichtung tätig/angestellt (extern) | 3, Es gibt sowohl interne als auch externe Fachkräfte
Wie viele Arbeitsprozente stehen für Infektionsprävention und -kontrolle zur Verfügung?	
Welche Aspekte sind in ihrer Einrichtung gewährleistet?	1, Schulungen und Fortbildungen zur Infektionsprävention und -kontrolle für Pflegefachpersonen und paramedizinische Personen (z.B. Physio, Ergo etc.) | 2, Schulungen und Fortbildungen zur Infektionsprävention und -kontrolle für ärztliches Personal (z.B. Haus- oder Heimärzt*innen) | 3, Entwicklung von Pflegestandards | 4, Erfassung von Bewohnenden, die mit multiresistenten Erregern kolonisiert/infiziert sind | 5, Zuständigkeit einer Person für die Meldung und das Management von Ausbrüchen | 6, Rückmeldung von Surveillance-Ergebnissen an das pflegerische und medizinische Personal der Einrichtung | 7, Beaufsichtigung/Kontrolle der Aufbereitung von Medizinprodukten und Pflegeartikeln (Desinfektion/Sterilisation) | 8, Entscheidung über Isolierungs- und Barrieremaßnahmen für Bewohnende, die mit multiresistenten Erregern besiedelt sind | 9, Regelmäßige Organisation, Kontrolle und Rückmeldung von Maßnahmen der Händehygiene in der Einrichtung | 10, Regelmäßige Organisation, Kontrolle und Rückmeldung über die Überprüfung oder Audits von etablierten Infektionspräventionsstrategien | 11, Möglichkeit, sich gegen die saisonale Influenza impfen zu lassen (Angebot für Bewohnende) | 12, Möglichkeit, sich gegen Sars-CoV2 boostern zu lassen (Angebot für Bewohnende) | 0, Keiner der oben aufgeführten Punkte
Gibt es in der Einrichtung eine Hygienekommission (intern oder extern)?	1, Ja | 0, Nein
Wie viele Treffen der Hygienekommission gab es im letzten Jahr? (2023)	
Kann die Einrichtung Hilfe und Expertise bei externen Fachexpert*innen oder Fachspezialist*innen in Infektionsprävention	1, Ja | 0, Nein

einholen? (z.B. Fachexpert*in eines lokalen Spitals oder des kantonsärztlichen Dienstes)	
Gibt es in der Einrichtung schriftliche Arbeitsanweisungen zu:	
dem Umgang mit MRSA und/oder anderen multiresistenten Erregern	1, Ja | 0, Nein
Massnahmen der Händehygiene	1, Ja | 0, Nein
dem Umgang mit Harnwegskathetern	1, Ja | 0, Nein
dem Umgang mit Gefäßkathetern	1, Ja | 0, Nein
den Umgang mit Sonden/Sondenernährung	1, Ja | 0, Nein
Umgang mit Ausbrüchen mit/bei respiratorischen Infektionen	1, Ja | 0, Nein
Umgang mit Ausbrüchen mit/bei gastrointestinalen Infektionen	1, Ja | 0, Nein
Umgang mit Exkrementen	1, Ja | 0, Nein
Gibt es ein Surveillancesystem zur Erfassung und Bewertung von Infektionen in der Einrichtung? (z.B. jährlicher zusammenfassender Bericht zur Häufigkeit von Harnwegsinfektionen, Atemwegsinfektionen, etc.)	1, Ja | 0, Nein
Falls ja, welches?	
Welche Methode zur Händehygiene wird am häufigsten in der Einrichtung angewendet, wenn die Hände nicht sichtbar verschmutzt sind?	1, Hygienische Händedesinfektion mit alkoholhaltigem Händedesinfektionsmittel | 2, Händewaschen mit Wasser und desinfizierender Seife | 3, Händewaschen mit Wasser und nicht-desinfizierender Seife
Falls verfügbar: Wie viele Liter alkoholischen Händedesinfektionsmittels wurden im letzten Jahr (2023) in der Einrichtung verbraucht?	
Wurden im letzten Jahr Schulungen/Fortbildungen zur Händehygiene für das Personal der Einrichtung durchgeführt?	1, Ja | 0, Nein
Masken: In welchen Situationen werden momentan in Ihrer Institution eine chirurgische Maske getragen? (mehrere Antworten möglich)	1, Immer innerhalb der Einrichtung | 2, In Kontakt mit Bewohnenden generell | 3, In Kontakt mit Bewohnenden, wenn diese respiratorische Symptome haben | 4, In Kontakt mit Bewohnenden, wenn man selber respiratorische Symptome hat | 5, In Kontakt mit anderen Mitarbeitenden, wenn man selber respiratorische Symptome hat | 6, In Ausbruchssituationen
Welche der folgenden Strukturen gibt es in der Einrichtung? (mehrere Antworten möglich)	1, Ein 'Antibiotika-Komitee'/eine Antibiotika-Kommission | 2, Regelmäßige, jährliche Fortbildungen über den angemessenen Einsatz von Antibiotika | 3, Schriftliche Richtlinien/Empfehlungen für den angemessenen Einsatz von Antibiotika | 4, Verfügbare Daten über den jährlichen Antibiotikaverbrauch, getrennt nach Wirkstoff-Gruppen | 5, Ein System, das medizinisches Personal an die Wichtigkeit mikrobiologischer Diagnostik zur Auswahl des geeigneten Antibiotikums hinweist bzw. erinnert | 6, Lokale (für Ihre Region) Resistenzprofile/Resistenzstatistiken sind der Einrichtung bzw. den verordnenden Ärzt*innen zugänglich | 7, Ein System, das die Zustimmung einer verantwortlichen Person erfordert, um Reserve-Antibiotika bzw. restriktiv zu verwendende Antibiotika zu verordnen | 8, Pharmakologische Beratung bei der Auswahl bzw. Verordnung von restriktiv zu verwendenden Antibiotika | 9, Therapierichtlinien, die auch eine Antibiotika-Liste beinhalten | 10, Rückmeldung der

	Antibiotikaverbrauchsdaten der Einrichtung an die Hausärzt*innen/verordnenden Ärzt*innen | 0, Keine der oben genannten Strukturen
Sofern es schriftliche Therapierichtlinien in der Einrichtung gibt, befassen sie sich mit:	
Atemwegsinfektionen?	1, Ja | 0, Nein
Harnwegsinfektionen?	1, Ja | 0, Nein
Wund- und Weichgewebeinfektionen?	1, Ja | 0, Nein
Gibt es ein System (z.B. ein Computerprogramm) zur Erfassung und Bewertung des Antibiotikaverbrauchs und zur Rückmeldung der Verbrauchsdaten in der Einrichtung?	1, Ja | 0, Nein
Gibt es ein System für die Surveillance (Erfassung und Bewertung) von multiresistenten Erregern in der Einrichtung? (z.B. eine jährliche zusammenfassende Übersicht hinsichtlich MRSA, Clostridium difficile etc.)	1, Ja | 0, Nein

Table S2. Resident questionnaire, adapted from the HALT-4 protocol (in German), Switzerland, 2024.

Field Label	Choices, Calculations, OR Slider Labels
Geburtsjahr	
Eintrittsjahr in die Einrichtung	
Geschlecht	1, Männlich | 0, Weiblich
RAI/BESA/PLAISIR Score	
Spitalaufenthalt in den letzten 3 Monaten	1, Ja | 0, Nein
Operation in den letzten 30 Tagen	1, Ja | 0, Nein
Mobilität	1, Mobil | 2, Rollstuhlpflichtig | 3, Bettlägerig
Harnwegskatheter	1, Ja | 0, Nein
Gefässkatheter	1, Ja | 0, Nein
Zeitliche und/oder örtliche Desorientierung	1, Ja | 0, Nein
Inkontinenz (Harn und/oder Stuhl)	1, Ja | 0, Nein
Protonen-Pumpen-Inhibitor (z.B. Pantozol oder Omeprazol)	1, Ja | 0, Nein
Dekubitus	1, Ja | 0, Nein
Andere Wunden	1, Ja | 0, Nein
Aktuelle antimikrobielle Therapie	1, Ja | 0, Nein

Table S3. Characteristics of participating and non-participating institutions from the random sample, Switzerland, 2024 (n=141).

	**Participating[a] n=49**	**Non-participating[a] n=92**	**p value**
Average resident age, median (range)	83 (71.6-87.0)	83.1 (42.2-87.1)	0.59
Number of beds, median (IQR)	61 (43-90)	48.5 (26-78)	<0.05
RAI/BESA/PLAISIR Score, median (IQR)	6.4 (3.1-9.3)	5.9 (2.7-10.5)	0.06
Language region			<0.01
German-speaking	31 (63.3%)	73 (79.3%)	
French-speaking	12 (24.5%)	18 (19.6%)	
Italian-speaking	6 (12.2%)	1 (1.1%)	
Qualified nursing staff per 1,000 nursing days	1.1 (0.6-2.5)	1.1 (0.3-4.0)	0.84

[a] N and %, if not stated otherwise

Table S4. Institutional characteristics of full sample between different language regions, Switzerland, 2024 (n=94).

	German[a] n=43	French[a] n=18	Italian[a] n=33	p-value
Type of facility				<0.001
Residential home	4 (9.3)	0	22 (66.7)	
General nursing home	29 (67.4)	12 (66.7)	9 (27.3)	
Mixed/other	10 (23.3)	6 (33.3)	2 (6.1)	
Nursing FTE/100 beds, median (IQR)	26.3 (16.3)	18.6 (6.8)	24.8 (9.8)	<0.01
Auxiliary nurse FTE/100 beds, median (IQR)	25.1 (6.6)	40.8 (10.7)	41.5 (12.9)	<0.001
Number of beds, median (IQR)	70 (69)	53.5 (17)	61 (32)	0.18
% of single beds, median (IQR)	90.6 (26.3)	86.7 (24.9)	93.9 (17.0)	0.18
Physician in charge				0.49
Personal family physician alone	19 (44.2)	6 (33.3)	17 (51.5)	
Employed by the facility alone	7 (16.3)	5 (27.8)	3 (9.1)	
Both	17 (39.5)	7 (38.9)	13 (39.4)	
Influenza vaccination residents, median (IQR)	55 (27)	81.5 (20.3)	77 (20)	<0.001
Influenza vaccination HCW, median (IQR)	10 (12)	29.5 (28)	20 (20)	<0.001
SARS-CoV-2 vaccination residents, median (IQR)	85 (12.5)	86 (49.8)	80 (22)	0.86
SARS-CoV-2 vaccination HCW, median (IQR)	70 (40.5)	25 (87.3)	55 (84)	0.79
IPC-trained HCW in facility	20 (46.5)	16 (88.9)	29 (87.9)	<0.001
% FTE IPC staff/100 beds, median (IQR)	11.4 (19.4)	16.5 (42.2)	10.4 (32.5)	0.70
Number of IPC activities, median (IQR)	8 (4)	9 (3)	8 (4)	0.27
IPC committee in place	18 (41.9)	3 (16.7)	16 (48.5)	0.08
Number of IPC guidelines, median (IQR)	7 (2)	5 (2)	7 (2)	0.06
Surveillance of HAI in place	3 (7.0)	5 (27.8)	8 (24.2)	0.06
Hand rub use, median liters/100 beds (IQR)	328 (231)	285 (147)	298 (298)	0.60

HCW, Healthcare Worker, IQR; Interquartile Range; FTE, Full-Time Equivalent; IPC, Infection Prevention and Control; HAI, Healthcare Associated Infection

[a] N and %, if not stated otherwise

Table S5. Healthcare associated infections and causing pathogens in Swiss long-term care residents, Switzerland, 2024 (n=169)

	**Urinary tract (n=73)**		**Respiratory (n=27)**			**COVID-19 (n=9)**		**Skin (n=26)**		**Gastrointestinal (n=6)**		**Ear, nose, throat (n=16)**			**Fever (n=1)**	**Other (n=11)**
	UTI-C (n=35)	UTI-P (n=38)	COLD-C (n=5)	LRTI-C (n=20)	PNEU-C (n=2)	COV-ASY (n=1)	COV-MM (n=8)	FUNG-C (n=8)	SKIN-C (n=18)	CDI-C (n=2)	GE-C (n=4)	CONJ-C (n=11)	EAR-C (n=4)	ORAL-C (n=1)	FUO-C (n=1)	
BCTOTH	2	16		8					6		1	3				1
CANALB		1							1							
CANGLA		1														
CLODIF										2						
CORSPP									1							
ENCFAE	4															
ENCNSP	1															
ENCOTH	1															
ESCCOL	23															
FUNOTH								4								
GPBNSP	1															
GPCOTH	1															
HAEINF			1													
KLEOTH	1															
KLEPNE	5															
LACSPP		1														
PRTMIR	1															1
PSEAER	1								1							1
STAAUR	1															
STANSP									1							
STRAGA																1
STROTH	1															
VIRCOV				1			7									
VIRNSP																1
VIROTH																1
VIRSAR				1			1									

Abbreviations: BCTOTH, Bacteria Other; CANALB, Candida albicans, CANGLA, Candida glabrata; CLODIF, Clostridium difficile; CORSPP; Corynebacterium species; ENCFAE, Enterococcus faecalis; ENCNSP; Enterococcus non specified; ENCOTH, other enterococci; ESCCOL, Escherichia coli; FUNOTH, other fungi; GPBNSP, grampositive bacilli, not specified; GPCOTH, other grampositive cocci; HAEINF, Haemophilus influenzae; KLEOTH, other Klebsiella; KLEPNE, Klebsiella pneumoniae; LACSPP, Lactobacillus species; PRTMIR, Proteus mirabilis; PSEAER, Pseudomonas aeruginosa; STAAUR, Staphylococcus aureus; STANSP, Staphylococcus non specified; STRAGA, Streptococcus agalactiae; STROTH, other streptococci; VIRCOV, SARS-CoV-2; VIRNSP, virus non specified; VIROTH, other virus; VIRSAR, SARS-Coronavirus

Table S6. Univariable and multivariable logistic regression regarding presence of healthcare-associated infections in Swiss long-term care residents (full sample), Switzerland, 2024 (n=164) For raw numbers, see supplementary Table S7; for mixed-effects models, see supplementary Table S8.

	Univariable analysis			Multivariable analysis		
	OR	95% CI	p-value	aOR	95% CI	p-value
**Resident-related factors**						
Age >85 years	1.03	0.76-1.42	0.83			
Male gender	1.38	1.00-1.89	**0.049**	1.22	0.87-1.70	0.25
Care dependency[b], median (IQR)	1.12	1.06-1.19	**<0.001**	1.00	0.93-1.07	0.89
Use of proton-pump inhibitor	1.27	0.93-1.73	0.13			
Disorientation	1.40	1.01-1.95	**0.046**	1.11	0.77-1.59	0.58
Wheelchair/bedridden	2.08	1.53-2.84	**<0.001**	1.36	0.94-1.95	0.10
Urinary catheter	3.65	2.41-5.36	**<0.001**	2.65	1.71-4.11	**<0.001**
Incontinence	2.13	1.45-3.23	**<0.001**	1.75	1.12-2.74	**0.01**
Chronic wound/decubital ulcer	2.31	1.63-3.23	**<0.001**	1.68	1.16-2.44	**0.006**
Hospitalisation/surgery last 3 months/90 days	1.93	1.27-2.85	**0.001**	1.64	1.08-2.49	**0.02**
**Institutional factors**						
Language						
German-speaking	Ref					
French-speaking	1.33	0.88-2.02	0.17			
Italian-speaking	1.20	0.85-1.71	0.30			
Type of facility						
Residential home	Ref					
General nursing home	0.96	0.65-1.40	0.82			
Mixed/other	1.10	0.72-1.70	0.66			
Nursing FTE/100 beds, median (IQR)	1.02	1.00-1.03	**0.033**	1.01	0.99-1.02	0.39
Auxiliary nurse FTE/100 beds, median (IQR)[c]	1.02	1.01-1.03	**0.001**	1.02	1.01-1.04	**0.002**
% of auxiliary nurses, median (IQR)	1.46	0.46-4.77	0.53			
Number of beds, median (IQR)	1.00	1.00-1.00	0.87			
% of single beds, median (IQR)	1.00	0.99-1.00	0.32			
Physician in charge						
Personal family physician alone	Ref			Ref		
Employed by the facility alone	1.54	0.98-2.43	0.06	1.64	1.03-2.62	**0.04**
Both	1.53	1.08-2.16	**0.02**	1.40	0.99-2.00	0.06
**IPC structures and parameters**						
Influenza vaccination residents, median (IQR)	1.00	0.99-1.00	0.33			

Influenza vaccination HCW, median (IQR)	1.01	0.99-1.01	0.39			
SARS-CoV-2 vaccination residents, median (IQR)	1.00	0.99-1.01	0.93			
SARS-CoV-2 vaccination HCW, median (IQR)	1.00	1.00-1.00	0.96			
IPC-trained HCW in facility	1.34	0.92-2.00	0.14			
% FTE IPC staff/100 beds, median (IQR)	1.00	1.00-1.00	0.43			
Number of IPC activities, median (IQR)	1.04	0.97-1.13	0.29			
IPC committee in place	1.61	1.18-2.20	**0.003**	1.38	0.98-1.93	0.06
Number of IPC guidelines, median (IQR)	1.03	0.92-1.15	0.60			
Surveillance of HAI in place	1.19	0.82-1.70	0.35			
Hand rub use, median liters/100 beds (IQR)	1.00	1.00-1.00	**0.04**			

OR, Odds Ratio; aOR; adjusted Odds Ratio; CI, Confidence Interval; HCW, Healthcare Worker, IQR; Interquartile Range; FTE, Full-Time Equivalent; IPC, Infection Prevention and Control; HAI, Healthcare Associated Infection; Ref, Reference

[a] If not stated otherwise; [b] Score ranging from 0 (<20 minutes of care per day) to 12 (>220 minutes of care per day); [c] Not included in the multivariable model due to high number of missing data (n=3,049)

Table S7. Raw numbers used for logistic regression analyses regarding HAI in Swiss long-term care residents (full sample). Note: numbers for institution-level factors partly differ from those in Table 1 due to the fact that all institutions are weighted equally in Table 1, whereas in this table institutions are weighted according to the number of residents, Switzerland, 2024 (n=7244)

	**No HAI[a] n=7,080**		**HAI[a] n=164**	
**Resident-related factors**				
Age >85 years	4,000	56.5	94	57.3
Male gender	2,127	30.0	61	37.2
Care dependency[b], median (IQR)	7	4	8	4
Use of proton-pump inhibitor	2,782	39.3	74	45.1
Disorientation	4,153	58.7	109	66.5
Wheelchair/bedridden	2,572	36.3	89	54.3
Urinary catheter	441	6.2	32	19.5
Incontinence	4,795	67.7	134	81.7
Chronic wound/decubital ulcer	1,075	15.2	48	29.3
Hospitalisation last 3 months/Surgery last 90 days	735	10.4	30	18.3
**Institutional factors**				
Language				
German-speaking	3,586	50.6	74	45.1
French-speaking	1,199	16.9	33	20.1
Italian-speaking	2,295	32.4	57	34.8
Type of facility				
Residential home	1,775	25.1	41	25.0
General nursing home	3,578	50.5	79	48.2
Mixed/other	1,727	24.4	44	26.8
Nursing FTE/100 beds, median (IQR)	24	14	26	17
Auxiliary nurse FTE/100 beds, median (IQR)	31	20	35	20
% of auxiliary nurses, median (IQR)	58	17	58	17
Number of beds, median (IQR)	93	125	95	105
% of single beds, median (IQR)	80	33	77	35
Physician in charge				
Personal family physician alone	3,086	43.6	55	33.5
Employed by the facility alone	1,056	14.9	29	17.7
Both	2,938	41.5	80	48.8
**IPC structures and parameters**				
Influenza vaccination residents, median (IQR)	70	28	70	30
Influenza vaccination HCW, median (IQR)	15	17	15	19
SARS-CoV-2 vaccination residents, median (IQR)	83	20	83	19
SARS-CoV-2 vaccination HCW, median (IQR)	68	75	55	81
IPC-trained HCW in facility	5,296	74.8	131	79.9
% FTE IPC staff/100 beds, median (IQR)	10	28	13	28
IPC committee in place	3,095	43.7	91	55.5
Number of IPC activities, median (IQR)	8	3	8	4
Number of IPC guidelines, median (IQR)	7	3	7	2
Surveillance of HAI in place	1,469	20.7	39	23.8
Alcoholic hand rub use, median liters/100 beds (IQR)	394	506	500	593

aOR; adjusted Odds Ratio; CI, Confidence Interval; HCW, Healthcare Worker, IQR; Interquartile Range; FTE, Full-Time Equivalent; IPC, Infection Prevention and Control; HAI, Healthcare Associated Infection; Ref, Reference

[a] N and %, if not stated otherwise; [b] Score ranging from 0 (<20 minutes of care per day) to 12 (>220 minutes of care per day)

Table S8. Univariable and multivariable mixed-effects (main analysis and sensitivity analysis) logistic regression regarding presence of healthcare-associated infections in Swiss long-term care residents (full sample), Switzerland, 2024 (n=164)

	**Mixed effects (main analysis)**						**Mixed effects (sensitivity analysis)**					
	**Univariable**			**Multivariable**			**Univariable**			**Multivariable**		
	**OR**	**95% CI**	**p-value**	**aOR**	**95% CI**	**p-value**	**OR**	**95% CI**	**p-value**	**aOR**	**95% CI**	**p-value**
**Resident-related factors**												
Age >85 years	1.03	0.76-1.42	0.83				1.05	0.76-1.43	0.79			
Male gender	1.38	1.00-1.89	**0.049**	1.22	0.87-1.70	0.25	1.37	0.99-1.90	0.055			
Care dependency[b], median (IQR)	1.12	1.06-1.19	**<0.001**	1.00	0.93-1.07	0.89	1.12	1.06-1.18	**<0.001**	1	0.94-1.07	0.94
Use of proton-pump inhibitor	1.27	0.93-1.73	0.13				1.27	0.93-1.75	0.14			
Disorientation	1.40	1.01-1.95	**0.046**	1.11	0.77-1.59	0.58	1.37	0.98-1.91	0.066			
Wheelchair/bedridden	2.08	1.53-2.84	**<0.001**	1.36	0.94-1.95	0.10	2.06	1.50-2.82	**<0.001**	1.37	0.95-1.98	0.09
Urinary catheter	3.65	2.41-5.36	**<0.001**	2.65	1.71-4.11	**<0.001**	3.67	2.45-5.50	**<0.001**	2.74	1.78-4.22	**<0.001**
Incontinence	2.13	1.45-3.23	**<0.001**	1.75	1.12-2.74	**0.01**	2.06	1.38-3.10	**<0.001**	1.73	1.12-2.69	**0.01**
Chronic wound/decubital ulcer	2.31	1.63-3.23	**<0.001**	1.68	1.16-2.44	**0.006**	2.3	1.63-3.26	**<0.001**	1.67	1.15-2.41	**0.007**
Hospitalisation last 3 months/Surgery last 90 days	1.93	1.27-2.85	**0.001**	1.64	1.08-2.49	**0.02**	1.95	1.30-2.94	**0.001**	1.67	1.10-2.54	**0.02**
**Institutional factors**												
Language												
German-speaking	Ref						Ref					
French-speaking	1.33	0.88-2.02	0.17				1.52	0.88-2.62	0.13			
Italian-speaking	1.20	0.85-1.71	0.30				1.27	0.81-2.00	0.30			
Type of facility												
Residential home	Ref						Ref					
General nursing home	0.96	0.65-1.40	0.82				0.98	0.61-1.59	0.94			
Mixed/other	1.10	0.72-1.70	0.66				1.08	0.60-1.93	0.81			
Nursing FTE/100 beds, median (IQR)[d]	1.02	1.00-1.03	**0.033**	1.01	0.99-1.02	0.39	1.01	0.99-1.03	0.17			
Auxiliary nurse FTE/100 beds, median (IQR)[d]	1.02	1.01-1.03	**0.001**	1.02	1.01-1.04	**0.002**	1.02	1.02-1.02	**0.02**	1.02	1.00-1.03	**0.02**
% of auxiliary nurses, median (IQR)[d]	1.46	0.46-4.77	0.53				1.44	0.32-6.42	0.63			
Number of beds, median (IQR)[d,e]	1.00	1.00-1.00	0.87				1.00	0.80-1.25	0.99			
% of single beds, median (IQR)[d,e]	1.00	0.99-1.00	0.32				0.92	0.75-1.12	0.40			
Physician in charge												
Personal family physician alone	Ref			Ref			Ref					
Employed by the facility alone	1.54	0.98-2.43	0.06	1.64	1.03-2.62	**0.04**	1.54	0.88-2.72	0.13			

Both	1.53	1.08-2.16	**0.02**	1.40	0.99-2.00	0.06	1.43	0.93-2.21	0.11			
**IPC structures and parameters**												
Influenza vaccination residents, median (IQR)[d,e]	1.00	0.99-1.00	0.33				0.95	0.78-1.15	0.59			
Influenza vaccination HCW, median (IQR)[d]	1.01	0.99-1.01	0.39				1.00	0.99-1.02	0.48			
SARS-CoV-2 vaccination residents, median (IQR)[d]	1.00	0.99-1.01	0.93				1.00	0.99-1.01	0.83			
SARS-CoV-2 vaccination HCW, median (IQR)[d]	1.00	1.00-1.00	0.96				1.00	1.00-1.00	0.92			
IPC-trained HCW in facility	1.34	0.92-2.00	0.14				1.38	0.86-2.22	0.18			
% FTE IPC staff/100 beds, median (IQR)[d]	1.00	1.00-1.00	0.43				1.00	1-00-1.00	0.24			
Number of IPC activities, median (IQR)[d]	1.04	0.97-1.13	0.29				1.02	0.93-1.13	0.67			
IPC committee in place	1.61	1.18-2.20	**0.003**	1.38	0.98-1.93	0.06	1.55	1.05-2.28	**0.03**	1.46	1.02-2.09	**0.04**
Number of IPC guidelines, median (IQR)[d]	1.03	0.92-1.15	0.60				1.01	0.88-1.16	0.88			
Surveillance of HAI in place	1.19	0.82-1.70	0.35				1.28	0.78-2.12	0.33			
Alcoholic hand rub, median liters/100 beds (IQR)[c,d,e]	1.00	1.00-1.00	**0.04**				1.19	0.96-1.47	0.10			

aOR; adjusted Odds Ratio; CI, Confidence Interval; HCW, Healthcare Worker, IQR; Interquartile Range; FTE, Full-Time Equivalent; IPC, Infection Prevention and Control; HAI, Healthcare Associated Infection; Ref, Reference

[a] If not stated otherwise

[b] Score ranging from 0 (<20 minutes of care per day) to 12 (>220 minutes of care per day)

[c] Not included in the multivariable model due to high number of missing data (n=3,049)

[d] Due to convergence issues, variables centered around a median of 0 in the random-effects model

[e] Due to convergence issues, variables rescaled to a standard deviation of 1 in the random-effects model, explaining the differences in the estimates and confidence intervals

Figure S1. Caterpillar plot showing residents with antibiotics (above) and with infection (below) across all participating institutions. Black dots represent estimates per institution, black vertical lines the 95% confidence interval. The blue line and the blue rhombus represent the overall prevalence, Switzerland, 2024 (n=7244)

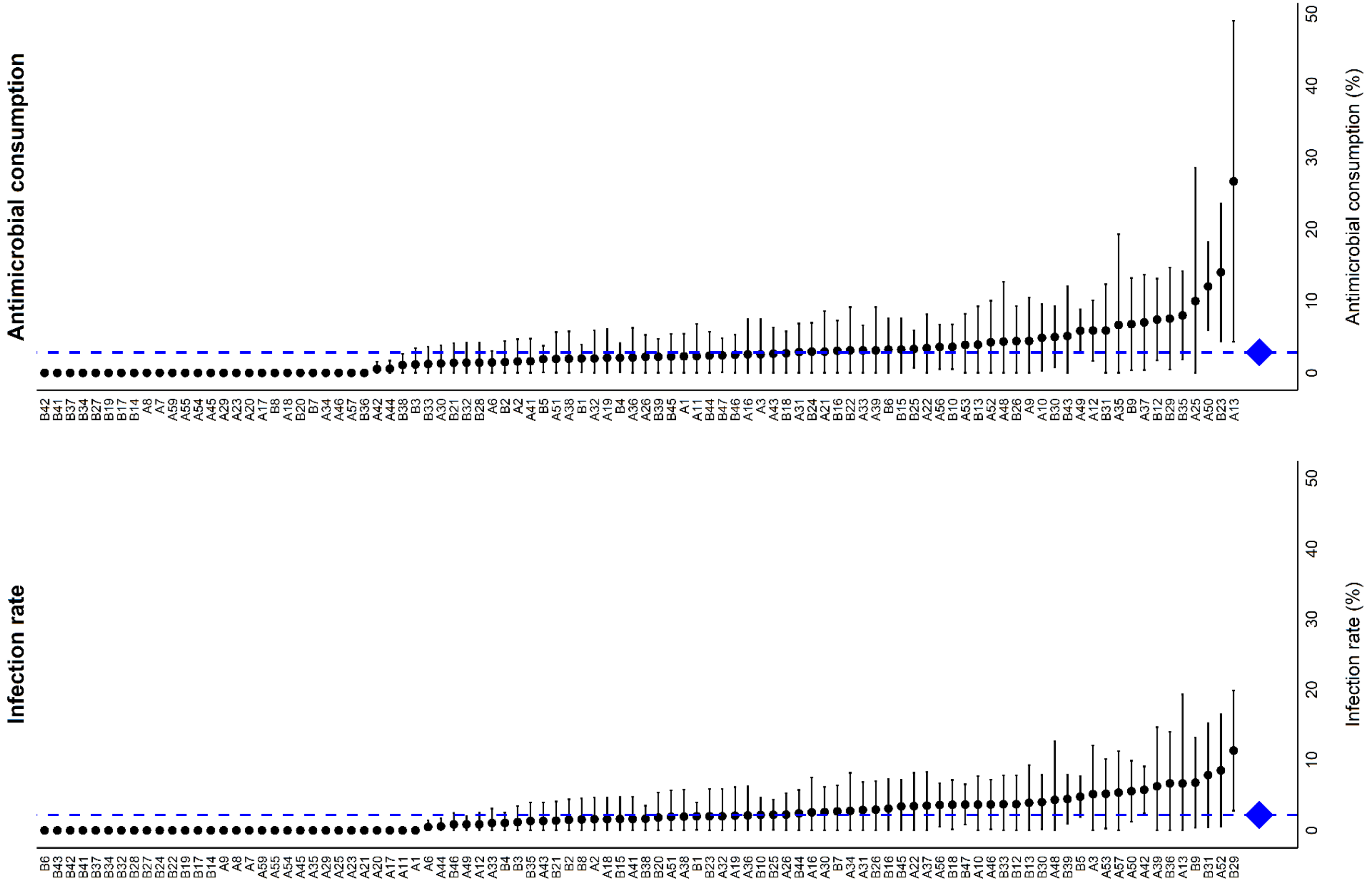

Supplement: Supplement [file 25-00221_FLURY_Supplement.pdf]
